# Anti-Interleukin-16 Neutralizing Antibody Treatment Alleviates Sepsis-Induced Cardiac Injury and Dysfunction via the Nuclear Factor Erythroid-2 Related Factor 2 Pathway in Mice

**DOI:** 10.1155/2021/6616422

**Published:** 2021-02-13

**Authors:** Jianwei Zhang, Zicong Yang, Zhishan Liang, Mengjie Wang, Changxing Hu, Chao Chang, Lei Shi, Qingwei Ji, Ling Liu

**Affiliations:** ^1^Department of Cardiology, Beijing Anzhen Hospital, Capital Medical University, Beijing Institute of Heart Lung and Blood Vessel Diseases, Beijing Key Laboratory of Precision Medicine of Coronary Atherosclerotic Disease, Clinical Center for Coronary Heart Disease, Capital Medical University, Beijing 100029, China; ^2^Department of Cardiology, The People's Hospital of Guangxi Zhuang Autonomous Region, Nanning, China; ^3^Department of Cardiology, Handan First Hospital, Handan, Hebei, China

## Abstract

Several interleukin (IL) members have been reported to participate in sepsis. In this study, the effects of IL-16 on sepsis-induced cardiac injury and dysfunction were examined, and the related mechanisms were detected. IL-16 expression in septic mice was first measured, and the results showed that both cardiac and serum IL-16 expression levels were increased in mice with sepsis induced by LPS or cecal ligation and puncture (CLP) compared with control mice. Then, IL-16 was neutralized, and the effects on lipopolysaccharide- (LPS-) induced cardiac injury were detected. The results showed that an anti-IL-16 neutralizing antibody (nAb) significantly reduced mortality and increased serum lactate dehydrogenase (LDH), creatine kinase myocardial bound (CK-MB), and cardiac troponin T (cTnT) levels while improving cardiac function in mice with LPS-induced sepsis. Neutralization of IL-16 also increased the activation of antioxidant pathways and the expression of antioxidant factors in septic mice while decreasing the activation of prooxidant pathways and the expression of prooxidants. Treatment with the anti-IL-16 nAb increased mitochondrial apoptosis-inducing factor (AIF) expression, decreased nuclear AIF and cleaved poly-ADP-ribose polymerase (PARP) expression, and decreased TUNEL-positive cell percentages in LPS-treated mice. Additionally, treatment with CPUY192018, the nuclear factor erythroid-2 related factor 2 (Nrf2) pathway, significantly increased mortality and reversed the above effects in mice treated with LPS and the anti-IL-16 nAb. Our results showed that the anti-IL-16 nAb regulates oxidative stress through the Nrf2 pathway and participates in the regulation of cardiac injury in septic mice. Neutralization of IL-16 may be a beneficial strategy for the prevention of cardiac injury and dysfunction in sepsis patients.

## 1. Introduction

Sepsis is a complex set of clinical syndromes that can lead to the failure of multiple vital organs. The most dangerous effects are cardiac injury and subsequent cardiac dysfunction, which is one of the most important complications affecting prognosis [1–3]. Due to its high incidence rate and its strong correlations with intensive care unit (ICU) admission and in-hospital mortality, sepsis was listed as a global health priority at the 2017 World Health Assembly [3, 4]. Further understanding of the pathogenesis of sepsis is crucial for sepsis treatment.

Interleukins (ILs) are a class of pluripotent cytokines that mediate multiple biological effects, and data from clinical and animal studies have demonstrated the involvement of multiple members of the interleukin family in sepsis. In an earlier study, circulating IL-26 levels were found to be significantly elevated in sepsis patients and to be correlated with severity and survival, and peritoneal inflammatory responses were found to be increased in septic mice treated with recombinant human IL-26 [5]. In another study, IL-33 deficiency aggravated sepsis-induced lung injury, while supplementation with IL-5 reversed this effect in IL-33 knockout mice, suggesting that IL-33 participates in the process of sepsis-induced lung injury by regulating IL-5 release [6]. In mice with cecal ligation and puncture- (CLP-) induced sepsis, treatment with IL-34 has been found to significantly reduce macrophage infiltration, protect against organ injury, and improve survival, while neutralization of IL-34 exerts the opposite effects [7]. Serum IL-38 levels are elevated in both septic adults and septic children, and neutralization of IL-38 significantly amplifies inflammatory responses and reduces survival in mice with both lipopolysaccharide- (LPS-) and CLP-induced sepsis [8].

IL-16, an important chemokine, is widely expressed in a variety of immune and nonimmune cells [9, 10]. In addition to regulating the infiltration and differentiation of immune cells, IL-16 has also been reported to regulate biological effects such as the inflammatory response and apoptosis [10–14]. To date, IL-16 has been shown to be involved in a variety of diseases, and its related mechanisms involve mainly the regulation of the inflammatory response [11–14]. IL-16 also plays roles in carotid atherosclerosis and angiotensin II- (Ang II-) induced cardiac fibrosis by amplifying inflammatory responses [15, 16]. However, the role of IL-16 in sepsis and its regulatory effects on oxidative stress are unknown. In this study, we determined whether IL-16 participates in sepsis-induced cardiac injury and dysfunction through the regulation of oxidative stress.

## 2. Experimental Materials and Methods

### 2.1. Mice, Treatments, and Sepsis Model Construction

**Figure 1:**
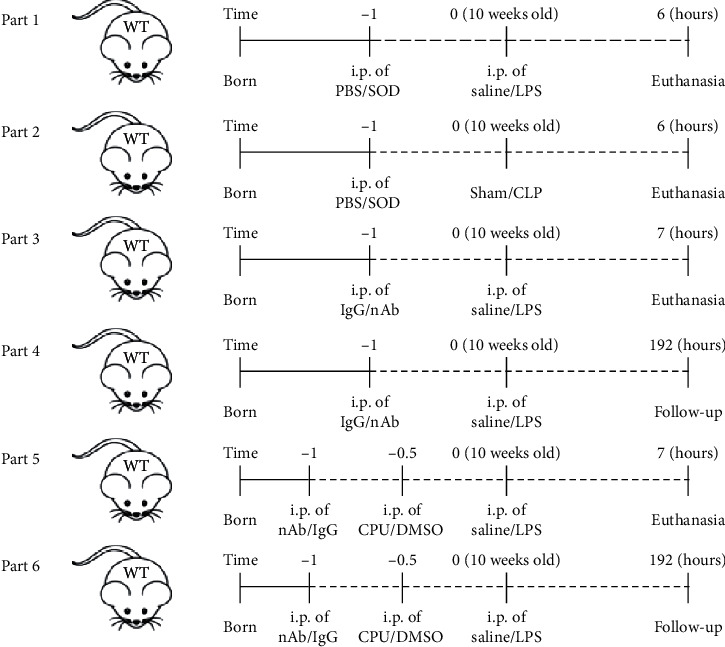
Establishment and pretreatment of the mouse sepsis model. WT: wild-type; PBS: phosphate-buffered saline; SOD: superoxide dismutase; LPS: lipopolysaccharide; CLP: cecal ligation and puncture; nAb: anti-IL-16 neutralizing antibody; CPU: CPUY192018; DMSO: dimethyl sulfoxide.

The use of the mice and the procedure for the experiment were approved by the Ethics Committee of the People's Hospital of Guangxi Zhuang Autonomous Region (approval no. 2015-16). Male wild-type (WT) mice purchased from Beijing Vital River Laboratory Animal Technology were used in this study. WT mice aged 10 weeks and weighing 25-27 g were used for the following experiments. First, mice were intraperitoneally injected (i.p.) with 10 mg/kg LPS (Sigma) or subjected to CLP for 6 hours. Some mice were given 400 *μ*/kg polyethylene glycol- (PEG-) superoxide dismutase (SOD) within 1 hour of sepsis [17]. Control mice received saline or underwent sham surgery and were treated with PBS (parts 1-2, *n* = 6). IL-16 expression was detected in these mice. Furthermore, WT mice were pretreated with 200 *μ*g of a mouse anti-IL-16 neutralizing antibody (nAb, BD Biosciences) or the same amount of isotype IgG (BD Biosciences) for 1 hour and then treated with LPS or saline for 6 hours (part 3, *n* = 10). Some mice were observed for 8 days (part 4, *n* = 9-13), and the mortality rates were recorded [16]. Additionally, mice that received the anti-IL-16 nAb or IgG were also given 50 *μ*l of dimethyl sulfoxide (DMSO, Sigma) or 5 mg/kg CPUY192018 (Sigma), a nuclear factor erythroid-2 related factor 2 (Nrf2) pathway inhibitor, and were treated with LPS (part 5, *n* = 10) [18]. The mortality rates of the different groups of mice were recorded during an 8-day follow-up (part 6, *n* = 9-12). The treatment of mice and the establishment of the sepsis model are shown in Figure 1.

In addition to LPS, CLP was used to establish a mouse sepsis model. According to the descriptions in previous literature, the general process was as follows: after anesthesia via inhalation of 1.5% isoflurane, the mice were laid flat on the operating table, and the entire abdomen of each rat was shaved. After alcohol disinfection, the abdominal skin was cut open, and the abdominal cavity was exposed. The cecum was found and ligated at half the distance between the distal pole and the base of the cecum, and then the cecum was punctured once from the mesenteric toward the antimesenteric direction using 26 G needles. The operation was considered complete after the skin was sutured and the abdominal skin was again disinfected [19].

### 2.2. Cell Culture Study

The mouse HL-1 cardiomyocytes used in this study were purchased from the American Type Culture Collection (ATCC, USA). HL-1 cardiomyocytes were plated in a 10 cm petri dish and cultured in the RPMI 1640 medium with 10% fetal bovine serum (both from Gibco, USA) in batches. HL-1 cardiomyocytes were transferred to 6 cm petri dishes once enough cells were obtained. After covering the 6 cm petri dish, the cells were synchronized for 24 hours and divided into 3 groups as follows: a group treated with 1 *μ*mol/ml LPS for 6 hours, a group treated with LPS+100 U/l PEG-SOD for 6 hours, and a control group treated with PBS [20, 21]. The culture medium was changed, and the stimulation was repeated every six hours. Twelve hours later, both the HL-1 cardiomyocytes and the culture supernatant were collected for IL-16 expression analysis.

### 2.3. Analysis of Cardiac Function

The mice were anesthetized with 1.5% isoflurane and laid flat on the operating table, and echocardiography was performed using a MyLab 30CV (Esaote) ultrasound system with a 15 MHz probe to measure left ventricular function in the mice. The left ventricular end-diastolic diameter (LVEDD), left ventricular end-systolic diameter (LVESD), left ventricular ejection fraction (LVEF, LVFS (%) = (LVEDD − LVESD)/LVEDD × 100%), and fractional shortening (FS) data were collected from 10 cardiac cycles and averaged.

### 2.4. Detection of Serum Levels of IL-16 and Cardiac Injury Markers

A mouse IL-16 enzyme-linked immunosorbent assay (ELISA) kit was purchased from BioLegend, and assay kits for cardiac injury markers, including lactate dehydrogenase (LDH), creatine kinase myocardial bound (CK-MB), and cardiac troponin T (cTnT), were purchased from Beyotime Biotechnology. Serum was collected after blood samples were centrifuged at 3000 × *g* for 20 minutes, and then the serum levels of IL-16, LDH, CK-MB, and cTnT were detected based on the manufacturer's instructions.

### 2.5. Separation of Mitochondria and Nuclei and Detection of Protein

Mitochondria were isolated from the left ventricle (LV) using a mitochondrial isolation kit (Cayman) as described in our previous study [22]. In brief, fresh LV tissue was lysed using a mitochondrial separation solution and then centrifuged at 700 × *g* for 10 minutes. Then, the supernatant was obtained and further centrifuged at 6000 × *g* for 10 minutes. The granular material at the bottom of the EP tube was collected as the mitochondrial fraction.

Nuclei were collected using a nuclear separation kit (Njjcbio) as previously described [22]. First, fresh LV tissue was lysed using the lysis buffer contained in the kit, and the homogenate was obtained and centrifuged at 1000 × *g* for 10 minutes. The granular material at the bottom of the EP tube was collected as the nuclear fraction.

Furthermore, western blotting was performed to detect protein expression as described in our previous study [23]. Briefly, mitochondria, nuclei, and LV tissue were lysed using the RIPA lysis buffer containing protease inhibitors and phosphatase inhibitors (both from Roche), and the total protein in each sample was collected after centrifugation (4000 × *g*) at 4°C and quantified using a BCA Protein Assay Kit (Thermo Fisher Scientific). Then, the proteins in 20 *μ*g total protein samples were separated by 10% gel electrophoresis and transferred to PVDF membranes (Millipore). After blocking with 5% nonfat milk, the membranes were incubated with the corresponding primary antibodies, which were used to label the target protein at 4°C overnight. The protein expression levels of IL-16, Nrf2, heme oxygenase 1 (HO-1), nicotinamide adenine nucleotide phosphate (NADPH) oxidase 2 (Nox2), Nox4, and GAPDH (antibodies all purchased from Abcam) in the LV; mitochondrial apoptosis-inducing factor (AIF) and cytochrome c oxidase IV (Cox IV) in mitochondria; and AIF, cleaved poly-ADP-ribose polymerase (PARP), and proliferating cell nuclear antigen (PCNA) in nuclei were labeled. Most of the blots originate from the same membrane in the figures. A small number of proteins are closer in molecular weight, so not all of them are in the same membrane. After incubation with the secondary antibody at room temperature for 1 hour, the blots were scanned using Odyssey (LI-COR Biosciences).

### 2.6. Analysis of Oxidative Stress Markers

In this study, appropriate kits (all purchased from Nanjing Jiancheng Bioengineering Institute) were used to detect serum SOD activity, glutathione (GSH) levels, NADPH oxidase activity, and malondialdehyde (MDA) levels according to the manufacturer's protocols. These molecules are all markers of oxidative stress.

### 2.7. Histological Analysis

After fixation, hearts were embedded in paraffin, cut into approximately 5 *μ*m thick sections, and mounted on slides. A mouse anti-4-hydroxynonenal (4-HNE) antibody (Abcam, UK) was used to perform 4-HNE staining to detect cardiac oxidative stress as described in our previous study [23]. Briefly, the sections were first deparaffinized and blocked and then incubated with the anti-4-HNE antibody (1 : 200) overnight at 4°C. Following incubation with the anti-rabbit HRP reagent for 1 h at 25°C and development using a peroxide-based substrate DAB kit (Genentech), the sections were dehydrated in ethanol and cleared in xylene. A commercially available kit was used to perform terminal deoxynucleotidyl transferase-mediated dUTP nick end labeling (TUNEL) staining (Millipore, USA) for analysis of cardiac apoptotic cells.

### 2.8. Data Statistics and Analysis

Data contained in this study are expressed as the mean ± SD and were analyzed by GraphPad Prism 8. When the data had a normal distribution, the differences between 2 groups were compared by Student's *t*-test, while differences in the mean during multiple (≥3) groups were determined using two-way ANOVA, followed by Tukey's multiple comparison test. While the data had an abnormal distribution, the differences were analyzed using a nonparametric test. The survival rates of mice during the 8-day follow-up were analyzed using the log-rank test. A *p* value less than 0.05 was considered statistically significant.

## 3. Results

### 3.1. Oxidative Stress Regulates IL-16 Expression in Sepsis

**Figure 2:**
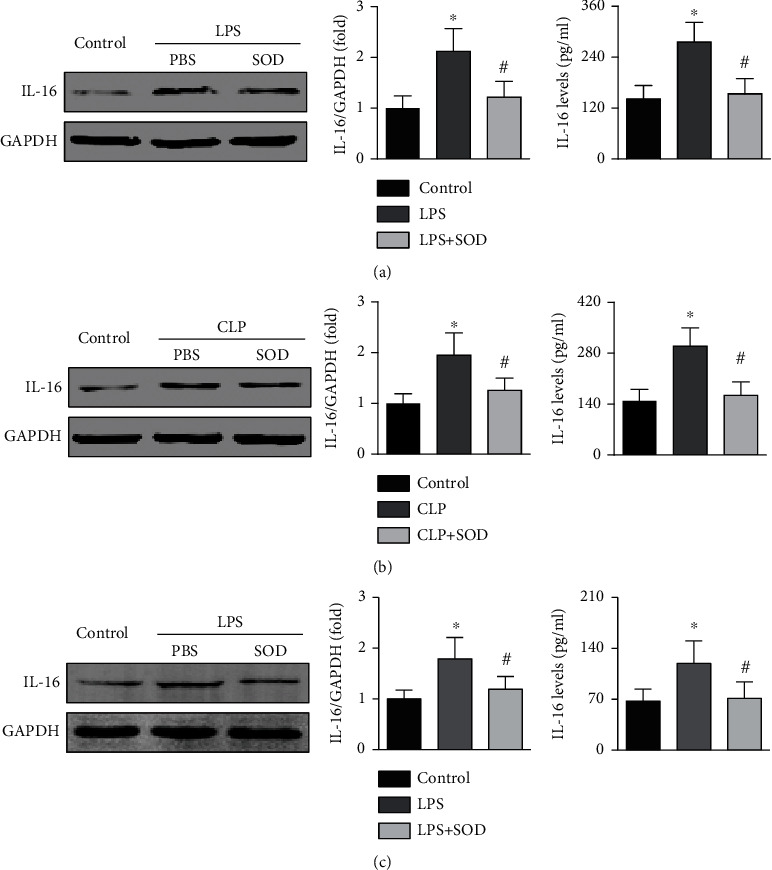
Effects of oxidative stress on IL-16 expression in sepsis. (a) Effects of SOD on cardiac IL-16 and serum IL-16 expression in LPS-treated mice (one-way ANOVA). (b) Effects of SOD on IL-16 expression in CLP-induced septic mice (nonparametric test). (c) Effect of SOD on IL-16 expression in LPS-treated HL-1 cardiomyocytes (nonparametric test). *n* = 6 in each group. ^∗^*p* < 0.05 vs. the control group. ^**#**^*p* < 0.05 vs. the LPS- or CLP-induced sepsis group. LPS: lipopolysaccharide; CLP: cecal ligation and puncture; SOD: superoxide dismutase.

In mice with LPS-induced sepsis, both cardiac and serum IL-16 expression levels were increased, but these increases were reversed by PEG-SOD treatment (Figure 2(a)). Similar trends were found for IL-16 expression in mice with CLP-induced sepsis (Figure 2(b)). LPS treatment also increased IL-16 expression in cardiomyocytes and IL-16 levels in the culture supernatant, while IL-16 levels were reduced in both the cardiomyocytes and the culture supernatant after PEG-SOD treatment (Figure 2(c)). In this section, the blots of IL-16 and GAPDH originated from the same membrane.

### 3.2. Anti-IL-16 nAb Treatment Alleviates Sepsis-Induced Cardiac Injury and Dysfunction in Mice

**Figure 3:**
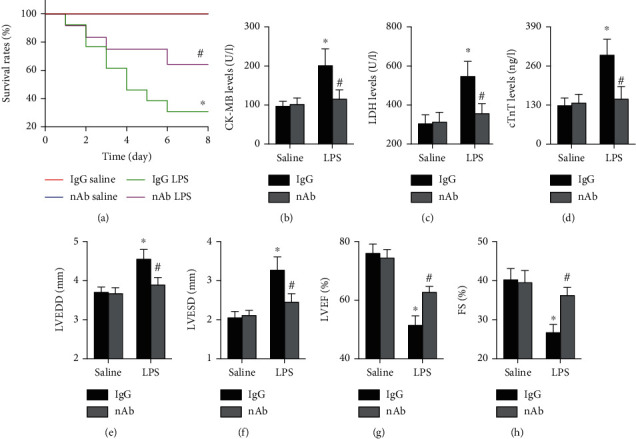
Effects of anti-IL-16 nAb on cardiac injury and function in septic mice. (a) Survival rates during the 8-day follow-up in each group (log-rank test). (b–d) Serum levels of oxidative stress markers in the four groups (two-way ANOVA). (e–h) Cardiac structure and function were determined by echocardiography (nonparametric test). *n* = 5-10 in each group. ^∗^*p* < 0.05 vs. the IgG saline group. ^**#**^*p* < 0.05 vs. the IgG LPS group. nAb: anti-IL-16 neutralizing antibody; LPS: lipopolysaccharide; LDH: lactate dehydrogenase; CK-MB: creatine kinase myocardial bound; cTnT: cardiac troponin T; LVEDD: left ventricular end-diastolic diameter; LVESD: left ventricular end-systolic diameter; LVEF: left ventricular ejection fraction; FS: fractional shortening.

The effects of the anti-IL-16 nAb on survival rates in each group were first analyzed, and the results showed that the anti-IL-16 nAb significantly improved survival rates in mice with LPS-induced sepsis but did not affect survival rates in saline-treated mice (Figure 3(a)). LPS-treated mice also showed higher serum CK-MB, LDH, and cTnT levels than control mice, and the LPS-induced increases were reversed by the anti-IL-16 nAb (Figures 3(b)–3(d)). In addition, neutralization of IL-16 reduced the LVEDD and LVESD and increased the percentages of both LVEF and FS in septic mice but did not affect LVEF or FS in mice without sepsis (Figures 3(e)–3(h)).

### 3.3. Anti-IL-16 nAb Treatment Reduces Oxidative Stress in Septic Mice

**Figure 4:**
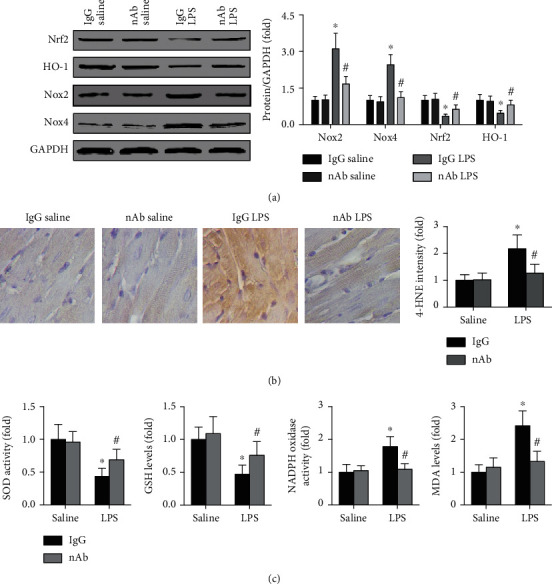
Effects of anti-IL-16 nAb on oxidative stress in septic mice. (a) Cardiac protein expression of Nrf2, HO-1, Nox2, Nox4, and GAPDH in each group was detected and analyzed (two-way ANOVA). (b) The cardiac 4-HNE intensity of the four groups was measured (nonparametric test). (c) The activities of SOD and NADPH oxidase and the levels of GSH and MDA in serum were determined (two-way ANOVA). *n* = 5 in each group. ^∗^*p* < 0.05 vs. the IgG saline group. ^#^*p* < 0.05 vs. the IgG LPS group. Nrf2: nuclear factor erythroid-2 related factor 2; HO-1: heme oxygenase 1; Nox2/4: nicotinamide adenine nucleotide phosphate oxidase 2/4; nAb: anti-IL-16 neutralizing antibody; LPS: lipopolysaccharide; 4-HNE: 4-hydroxynonenal; SOD: superoxide dismutase; GSH: glutathione; NADPH: nicotinamide adenine nucleotide phosphate; MDA: malondialdehyde.

Furthermore, the effects of the anti-IL-16 nAb on oxidative stress were detected. The results showed that neutralization of IL-16 increased cardiac Nrf2 and HO-1 expression while decreasing cardiac Nox2 and Nox4 levels in septic mice (Figure 4(a)). In this section, the blots of Nrf2, HO-1, and Nox4 did not originate from the same membrane because their molecular weights are similar to those of Nox2 or GAPDH or Nrf2. Lower cardiac 4-HNE staining intensity was observed in anti-IL-16 nAb-treated septic mice than in IgG-treated septic mice (Figure 4(b)). In addition, anti-IL-16 nAb treatment also increased SOD activity and GSH levels while decreasing Nox activity and MDA levels in serum from septic mice (Figure 4(c)).

### 3.4. Anti-IL-16 nAb Treatment Decreases AIF-Related Cardiomyocyte Apoptosis in Septic Mice

**Figure 5:**
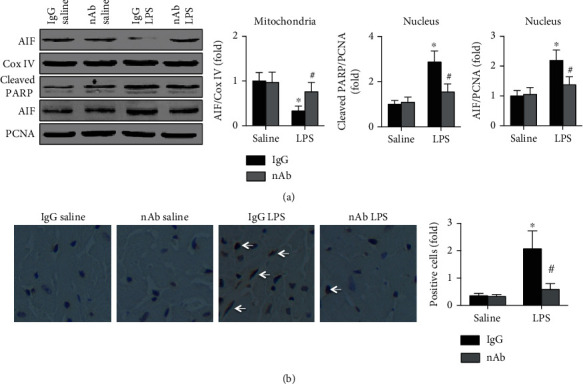
Effects of IL-16 nAb on AIF expression in LPS-treated mice. (a) Cardiac mitochondrial AIF and Cox IV expression and cardiac nuclear AIF, cleaved PARP, and PCNA expression in each group were detected (two-way ANOVA). (b) TUNEL-positive cells in the four groups were analyzed (nonparametric test). *n* = 5 in each group. ^∗^*p* < 0.05 vs. the IgG saline group. ^#^*p* < 0.05 vs. the IgG LPS group. AIF: apoptosis-inducing factor; Cox IV: cytochrome c oxidase IV; PARP: poly-ADP-ribose polymerase; PCNA: proliferating cell nuclear antigen; nAb: anti-IL-16 neutralizing antibody; LPS: lipopolysaccharide.

AIF expression was detected in both mitochondria and nuclei from LV tissue. The results showed that neutralization of IL-16 significantly increased mitochondrial AIF expression while decreasing nuclear AIF and cleaved PARP expression in septic mice (Figure 5(a)). The blots of AIF and Cox IV in the mitochondria originated from the same membrane, and the blots of cleaved PARP, AIF, and PCNA in the nucleus originated from the same membrane. In addition, fewer TUNEL-positive cells were observed in nAb- and LPS-treated mice than in IgG- and LPS-treated mice (Figure 5(b)). The anti-IL-16 nAb did not affect either the AIF expression or the number of TUNEL-positive cells (Figures 5(a) and 5(b)).

### 3.5. CPUY192018 Treatment Reverses the Effects of Anti-IL-16 nAb Treatment on Sepsis-Induced Cardiac Injury

**Figure 6:**
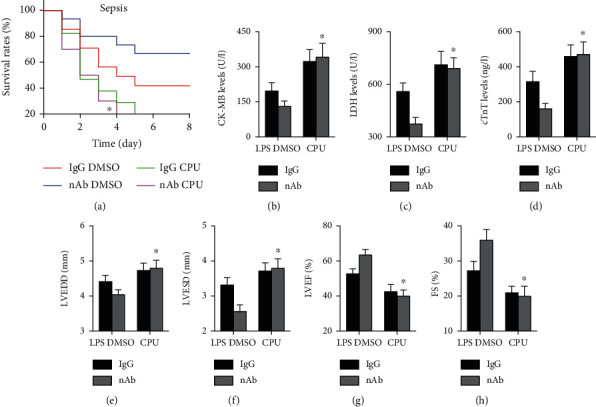
Effects of CPUY192018 on cardiac injury in septic mice. (a) Survival rates were measured for each group (log-rank test). (b–d) Serum levels of cardiac injury markers, including CK-MB, LDH, and cTnT, were measured (two-way ANOVA). (e–h) Cardiac structure and function in each group were measured (two-way ANOVA). *n* = 5 in each group. ^∗^*p* < 0.05 vs. the nAb DMSO+LPS group. LPS: lipopolysaccharide; DMSO: dimethyl sulfoxide; CPU: CPUY192018; nAb: anti-IL-16 neutralizing antibody; LDH: lactate dehydrogenase; CK-MB: creatine kinase myocardial bound; cTnT: cardiac troponin T; LVEDD: left ventricular end-diastolic diameter; LVESD: left ventricular end-systolic diameter; LVEF: left ventricular ejection fraction; FS: fractional shortening.

The effects of CPUY192018 on survival rates were determined, and the results showed that CPUY192018 treatment significantly increased mortality in anti-IL-16 nAb-treated septic mice (Figure 6(a)). Serum LDH, CK-MB, and cTnT levels were all elevated by CPUY192018 in anti-IL-16 nAb-treated septic mice (Figures 6(b)–6(d)). Higher LVEDD and LVESD and lower LVEF and FS percentages were observed in CPUY192018-treated septic mice than in CPUY192018-treated mice without LPS-induced sepsis (Figures 6(e)–6(h)).

### 3.6. The Nrf2 Pathway Mediates the Antioxidative and Antiapoptotic Effects of Anti-IL-16 nAb Treatment in Septic Mice

**Figure 7:**
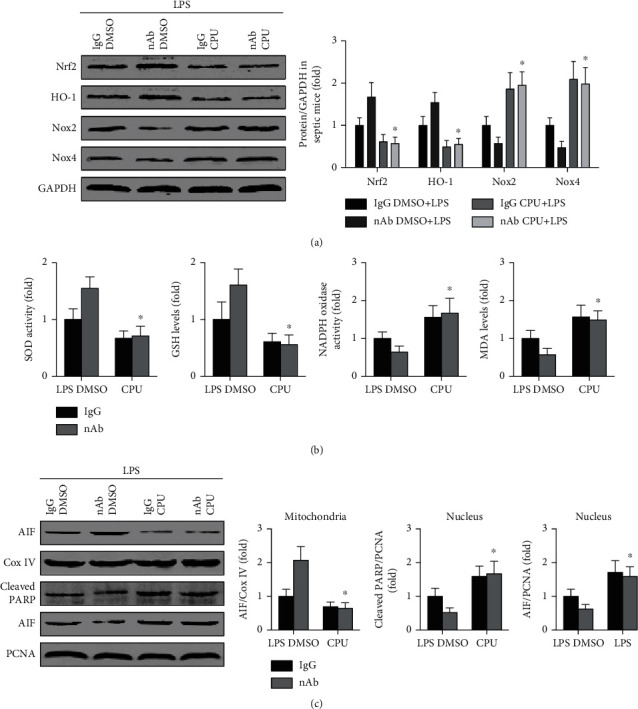
Effects of CPUY192018 on oxidative stress and cardiomyocyte apoptosis in septic mice. (a) Nrf2, HO-1, Nox2, Nox4, and GAPDH expression in LV tissue was detected (two-way ANOVA). (b) Serum activity of SOD and NADPH oxidase and levels of GSH and MDA were measured (two-way ANOVA). (c) AIF and Cox IV expression in mitochondria and AIF, cleaved PARP, and PCNA expression in nuclei were analyzed (two-way ANOVA). *n* = 5 in each group. ^∗^*p* < 0.05 vs. the nAb DMSO+LPS group. Nrf2: nuclear factor erythroid-2 related factor 2; HO-1: heme oxygenase 1; Nox2/4: nicotinamide adenine nucleotide phosphate oxidase 2/4; nAb: anti-IL-16 neutralizing antibody; CPU: CPUY192018; LPS: lipopolysaccharide; SOD: superoxide dismutase; GSH: glutathione; NADPH: nicotinamide adenine nucleotide phosphate; MDA: malondialdehyde; AIF: apoptosis-inducing factor; Cox IV: cytochrome c oxidase IV; PARP: poly-ADP-ribose polymerase; PCNA: proliferating cell nuclear antigen.

Treatment with CPUY192018 decreased Nrf2 and HO-1 levels and increased Nox2 and Nox4 expression in anti-IL-16 nAb-treated septic mice (Figure 7(a)). The anti-IL-16 nAb-mediated reductions in Nox and MDA levels and the increases in SOD activity and GSH levels in mice with LPS-induced sepsis were reversed by CPUY192018 treatment (Figure 7(b)). Additionally, inhibition of the Nrf2 pathway with CPUY192018 decreased cardiac mitochondrial AIF expression while increasing cardiac nuclear AIF and cleaved PARP expression in septic mice pretreated with the anti-IL-16 nAb (Figure 7(c)).

## 4. Discussion

In this study, we examined the effect of IL-16 on sepsis-induced cardiac injury and dysfunction and focused on oxidative stress to elucidate the possible mechanisms. We found, for the first time, that both cardiac and serum IL-16 expression levels were elevated in both LPS- and CLP-induced mouse sepsis models. Moreover, neutralization of IL-16 significantly improved survival rates, ameliorated cardiac dysfunction, reduced the expression of multiple cardiac injury markers, alleviated oxidative stress, and protected cardiomyocytes from apoptosis. Inhibition of the Nrf2 pathway with CPUY192018 significantly reversed the above effects of the anti-IL-16 nAb in septic mice.

The mechanisms by which sepsis induces cardiac injury and dysfunction are very complex; data from animal studies have revealed that a variety of complex pathological effects, including inflammation, oxidative stress, and autophagy, are involved [24–26]. In recent years, an increasing number of studies have focused on oxidative stress because sepsis can lead to severe oxidative stress in a variety of different organs and tissues [26–28]. Our study revealed that the expression of IL-16 was significantly increased in septic mice and LPS-treated cardiomyocytes and that this upregulation could be reversed by SOD, a common antioxidant. These results suggest that IL-16 expression is regulated by oxidative stress levels during sepsis, although IL-16 release in other models is regulated by inflammatory responses. In a subsequent LPS-induced mouse sepsis model, neutralization of IL-16 significantly increased survival, reduced the expression of multiple markers of cardiac injury, and improved cardiac function. These data suggest that reducing the expression of IL-16 can attenuate sepsis-induced cardiac injury and dysfunction. Considering that oxidative stress is an important factor affecting the secretion of IL-16, we speculate that IL-16 may participate in the process of myocardial injury and dysfunction by regulating the level of oxidative stress.

Under normal physiological conditions, prooxidant and antioxidant substances maintain a dynamic balance, which is essential for the maintenance of normal physiological activities [28, 29]. Under the action of doxorubicin, Ang II, and other external pathogenic factors, this stable equilibrium relationship is broken, as evidenced by reductions in the levels of antioxidants, including NRf2, HO-1, and SOD, and increases in the levels of oxygen-promoting substances, including Nox2, Nox4, and MDA [30, 31]. To determine whether IL-16 is involved in sepsis-induced cardiac injury through the regulation of oxidative stress, pathways and markers related to oxidative stress were detected. The results showed that neutralization of IL-16 significantly reversed sepsis-induced oxidative stress imbalance in both the heart and the serum. These results support our hypothesis and provide the first evidence that IL-16 regulates oxidative stress in addition to inflammatory responses.

There are also regulatory effects among oxidative stress-related pathways. One such pathway, the Nrf2 signaling pathway, regulates the physiological response to oxidative stress and is very important for the maintenance of cellular redox balance. In a recent study, Kim et al. reported that sodium butyrate promoted inactivation of the nuclear factor kappa-B (NF-*κ*B) pathway through the Nrf2 pathway, upregulated SOD levels, and inhibited Nox2 expression in a mouse Alzheimer's disease model [32].

In a mouse orthotopic liver transplantation model, Ke et al. found that deletion of the Nrf2 pathway significantly reduced HO-1 pathway activation [33]. In an earlier study, the authors reported that Nrf2 deficiency elevated ROS levels by upregulating both Nox2 expression and Nox4 expression [34]. These results indicate that the Nrf2 signaling pathway regulates the activation of other oxidative stress-related pathways and the expression of oxidative stress-related markers during oxidative stress regulation, which means that the Nrf2 pathway plays a leading role in the process of oxidative stress. To investigate whether the protective effect of the anti-IL-16 nAb against sepsis-induced cardiac injury was mediated by the Nrf2 pathway, CPUY192018 was used to inhibit the Nrf2 pathway in mice with LPS-induced sepsis. The results showed that treatment with CPUY192018 significantly decreased cardiac HO-1 levels, increased Nox2 and Nox4 expression, and aggravated oxidative stress imbalance in septic mice with neutralized IL-16. These results are consistent with previous conclusions and suggest that IL-16 regulates downstream oxidative stress through the Nrf2 pathway.

In a mouse sepsis model, increased oxidative stress is often accompanied by increased cardiomyocyte apoptosis, and reversing the increase in oxidative stress significantly attenuates cardiomyocyte apoptosis [34, 35]. This suggests that oxidative stress is an important cause of cardiomyocyte apoptosis in the context of sepsis. Large numbers of apoptotic cardiomyocytes are found in septic animals; such extensive apoptosis can lead to declines in cardiac function. However, reducing cardiomyocyte apoptosis can significantly reverse cardiac dysfunction, suggesting that sepsis-induced excessive apoptosis of cardiomyocytes might be the most fundamental cause of cardiac injury [36]. AIF, which exists mainly in the mitochondrial inner membrane and is less prevalent or nonexistent in the nucleus, is one of the important factors mediating non-Caspase-dependent apoptosis [22]. Under stimulation by external pathological factors, AIF is released in massive quantities by mitochondria and transferred to the nucleus through the cytoplasm [22]. Increases in nuclear AIF levels promote apoptosis, which can act through a feedback mechanism to promote the nuclear expression of cleaved PARP, a DNA damage repair protein, to protect against apoptosis [22]. To further explore the mechanisms, cardiomyocyte apoptosis was detected in septic mice. The results showed that inhibition of the Nrf2 pathway with CPUY192018 significantly promoted the transfer of mitochondrial AIF to the nucleus and increased the expression of nuclear cleaved PARP in septic mice that were pretreated with the anti-IL-16 nAb. In addition, more TUNEL-positive cells were found in septic mice with IL-16 neutralization than in septic mice without neutralization. These results indicate that inhibition of the Nrf2 pathway can significantly reverse the protective effect of the anti-IL-16 nAb against AIF-mediated cardiomyocyte apoptosis in septic mice.

In conclusion, our study demonstrates, for the first time, that neutralization of IL-16 can upregulate the Nrf2 pathway, reduce oxidative stress, inhibit the transfer of AIF from the mitochondria to the nucleus, and thus reduce cardiomyocyte apoptosis, alleviate cardiac injury, and improve cardiac function in septic mice.

## Data Availability

We declare that the materials described in the manuscript, including all relevant raw data, will be freely available to any scientist wishing to use them for noncommercial purposes without breaching participant confidentiality.
